# Hiatoplasty with Crura Buttressing versus Hiatoplasty Alone during Laparoscopic Sleeve Gastrectomy

**DOI:** 10.1155/2017/6565403

**Published:** 2017-11-12

**Authors:** Andrea Balla, Silvia Quaresima, Pietro Ursi, Ardit Seitaj, Livia Palmieri, Danilo Badiali, Alessandro M. Paganini

**Affiliations:** ^1^Department of General Surgery and Surgical Specialties “Paride Stefanini”, Sapienza University of Rome, Rome, Italy; ^2^Department of Internal Medicine and Medical Specialties, Sapienza University of Rome, Rome, Italy

## Abstract

**Introduction:**

In obese patients with hiatal hernia (HH), laparoscopic sleeve gastrectomy (LSG) with cruroplasty is an option but use of prosthetic mesh crura reinforcement is debated. The aim was to compare the results of hiatal closure with or without mesh buttressing during LSG.

**Methods:**

Gastroesophageal reflux disease (GERD) was assessed by the Health-Related Quality of Life (GERD-HRQL) questionnaire before and after surgery in two consecutive series of patients with esophageal hiatus ≤ 4 cm^2^. After LSG, patients in group A (12) underwent simple cruroplasty, whereas in group B patients (17), absorbable mesh crura buttressing was added.

**Results:**

At mean follow-up of 33.2 and 18.1 months for groups A and B, respectively (*p* = 0.006), the mean preoperative GERD-HRQL scores of 16.5 and 17.7 (*p* = 0.837) postoperatively became 9.5 and 2.4 (*p* = 0.071). In group A, there was no difference between pre- and postoperative scores (*p* = 0.279), whereas in group B, a highly significant difference was observed (*p* = 0.002). The difference (Δ) comparing pre- and postoperative mean scores between the two groups was significantly in favor of mesh placement (*p* = 0.0058).

**Conclusions:**

In obese patients with HH and mild-moderate GERD, reflux symptoms are significantly improved at medium term follow-up after cruroplasty with versus without crura buttressing during LSG.

## 1. Introduction

Hiatal hernia (HH) and gastroesophageal reflux disease (GERD) are often concomitant conditions and are more frequent in obese than in nonobese patients [1]. The prevalence of HH in obese patients ranges from 5 to 50% [2–4]. Several factors contribute to HH development: increased intrabdominal pressure, increased visceral fat, congenital gastroesophageal junction anomalies, and chronic reflux [5, 6]. Patients who are candidates to bariatric surgery often present with GERD, with a 50–70% incidence rate [5, 6]. The treatment of choice in obese patients with GERD symptoms is laparoscopic Roux-en-Y gastric bypass (LRYGB) [7, 8]. However, patients' demand is more frequently in favor of laparoscopic sleeve gastrectomy (LSG), which is felt by patients to be associated with lower morbidity and does not require long-term vitamins and elemental nutrient support; the cost of which is not covered by the health system.

LSG is currently the most common surgical procedure for the treatment of morbid obesity, both in Europe and in the USA [9, 10], but its results in relation to GERD symptoms are controversial [11–13]. Some authors report improved symptoms after weight loss, while others report de novo GERD in patients who were asymptomatic before surgery [11–13].

When a HH is present, simultaneous repair is possible and recommended during LSG [14, 15]. Two techniques have been proposed: after reduction in the peritoneal cavity of the herniated stomach and abdominal esophagus, the diaphragmatic crura are sutured together anteriorly or, preferably, posteriorly (simple cruroplasty) [16]. After cruroplasty, the diaphragmatic crura may be buttressed with nonabsorbable mesh, although the use of this type of mesh has been reported to be at a higher risk of esophageal stricture, erosion, or perforation [17]. Alternatively, it is possible to reinforce the diaphragmatic crura with an absorbable synthetic patch fixed by absorbable tacks [16, 18]. This patch causes the development of granulation tissue and neoangiogenesis at the level of the crura, and it is completely reabsorbed after six to eight months [16, 18]. The fibrotic tissue that remains after mesh absorption is meant to reduce the risk of HH recurrence [16, 18].

The aim of this retrospective study was to compare the results of simple posterior cruroplasty versus reinforced cruroplasty during LSG, in terms of GERD symptoms, in patients with an esophageal hiatal area measuring ≤ 4 cm^2^.

## 2. Materials and Methods

This is a retrospective analysis of prospectively collected data. Institutional review board approval and informed consent from all individual participants included in the study were obtained. From October 2006 to April 2017, 157 obese patients underwent LSG in the “Clinica Chirurgica e Tecnologie Avanzate,” Department of General Surgery and Surgical Specialties “Paride Stefanini,” Policlinico Umberto I, “Sapienza” University of Rome, Italy, a unit that was mainly devoted to minimally invasive oncologic surgery. Laparoscopic hiatoplasty in obese patients was performed since May 2011.

At admission, the patients were assessed according to National and International Guidelines [19], as previously reported [20], and included in the study. Exclusion criteria were body mass index (BMI) < 35 kg/m^2^, patients at prohibitively high risk for general anesthesia and induction of pneumoperitoneum, hiatus area > 4 cm^2^, sweet eaters, pregnancy, alcohol or drug consumption, and severe psychiatric disorders. The HH size was evaluated preoperatively by endoscopy. The esophageal hiatus area was calculated intraoperatively as the area of the rhomboid space that is crossed by the esophagus (d_1_ × d_2_/2) [16].

Patients with HH and a hiatus area measuring ≤ 4 cm^2^ underwent either LSG, hernia reduction, and simple cruroplasty (group A) prior to absorbable patch availability in our hospital or LSG, hernia reduction, and cruroplasty reinforced by absorbable patch placement (group B), subsequently. GERD symptoms were assessed by a Health-Related Quality of Life (GERD-HRQL) questionnaire before and after surgery [21]. Postoperatively, HH recurrence was evaluated by barium swallow.

### 2.1. Questionnaire

The gastroesophageal reflux disease-Health-Related Quality of Life (GERD-HRQL) is a patient self-rating questionnaire comprising 10 questions that specifically investigate GERD symptoms, each one with a score from 0 (absence of symptoms) to 5 (severe symptoms), for a total score that may range from 0 to 50 [21].

### 2.2. Surgical Technique

Surgery was performed with the patient in supine, steep anti-Trendelenburg position, and operative table tilted with the patient's right side down, in order to improve exposure of the operative field. The surgeon stood in between the patient's legs.

Pneumoperitoneum was established with an optical trocar and a 30° optic. The first 12 mm trocar was inserted left of the midline, at a point at the junction between the upper two thirds and the lower third of the line between the xiphoid process and umbilicus. The second and third 12 mm trocars were placed along the left and right pararectal lines, two fingerbreadths below the costal arches. A fourth 5 mm trocar was placed in a subxiphoid position, right of the midline, and a fifth 5 mm trocar was placed in the left hypochondrium, along the anterior axillary line [20]. The first step of the procedure was the division of the gastric greater curvature attachments to the greater omentum by means of a radiofrequency (LigaSure™ tissue fusion, Covidien, Mansfield, Massachusetts, USA) or ultrasonic (Ultracision, harmonic scalpel, Ethicon Endo-Surgery, Cincinnati, Ohio, USA) device, starting 5 cm from the pylorus and proceeding orally up to the angle of His. The left and right bundles of the right crus of the esophageal hiatus were exposed, and the upper stomach, gastroesophageal junction, and lower esophagus were circumferentially mobilized until the stomach and approximately 4 cm of intra-abdominal esophagus were reduced in the abdominal cavity. LSG was then performed with a linear stapler (Echelon Flex Powered Endopath, Ethicon Endo-Surgery, Johnson & Johnson, Cincinnati, OH), beginning with 2 green or black cartridges on the antrum, followed by 2–4 green cartridges on the body and fundus, all reinforced by Seam-guard® (Gore & Associates, Inc., Newark, Delaware, USA), starting 5 cm proximal to the pylorus under guidance of an orogastric 36 Fr bougie laid along the smaller curvature of the stomach. After sleeve creation, in group A, the left and right bundles of the right crus were sutured together posteriorly to the esophagus with the bougie in place, with 2 or 3 stitches of a nonabsorbable 2.0 braided polyester suture (Ethibond, Ethicon, Cincinnati, Ohio, USA). In group B, after hiatal closure was completed, a “U” shaped polyglycolic acid-trimethylene carbonate absorbable synthetic patch (mesh Bio-A®, Gore & Associates, Inc., Newark, Delaware, USA) was fixed with absorbable tacks (AbsorbaTack 5 mm Fixation Device, Covidien, Mansfield, Massachusetts, USA) to the crura. Care was taken to avoid placing mesh and tacks in contact with the esophagus. After removing the bougie, the resected portion of the stomach was extracted after enlarging the trocar site above the umbilicus.

### 2.3. Measures of Outcomes

The primary endpoint was an improvement of GERD symptoms, as recorded by the GERD-HRQL questionnaire score evaluation before and after surgery in the two groups. Secondary endpoints were operative time, conversion rate, intra- and postoperative complications (according to Clavien-Dindo classification [22]), hospital stay, mortality, postoperative BMI, and percentage excess body mass index loss (%EBMIL). In case of leaks, these were classified according to the International Sleeve Gastrectomy Expert Panel Consensus Statement [15].

### 2.4. Statistical Analysis

Statistical analysis was performed using the *t*-test, and data are presented as mean ± standard deviation (SD). Fisher's exact test was used to evaluate the difference within groups. A probability (*p*) value lower than 0.05 was considered statistically significant. All statistical analyses were carried out with SPSS software 19.0 (SPSS Inc., Chicago, Illinois, USA).

## 3. Results and Discussion

### 3.1. Results

Out of a total series of 157 patients who underwent LSG, 12 patients with HH underwent simple cruroplasty from May 2011 to March 2014 (group A), and 21 patients with HH underwent cruroplasty with mesh buttressing from April 2014 to April 2017 (group B). Among group B patients, 1 had an esophageal hiatus area measuring more than 4 cm^2^ and was excluded from the present analysis, and 3 patients were lost at follow-up, leaving 12 and 17 patients in groups A and B, respectively, who were available for the study.

Tables 1 and 2 show the patients' characteristics. Before surgery, the only statistically significant difference that was observed between the two groups was the rate of preoperative diagnosis of HH, more frequent in group B (*p* = 0.0382) (Table 2). However, no significant differences between the two groups were observed in the incidence of GERD symptoms and esophagitis (Table 2).

Mean operative time, in groups A and B, was 195.4 ± 51.9 minutes (range 130–300 minutes) and 184.3 ± 39.09 minutes (range 130–300 minutes), respectively (*p* = 0.5326). At surgery, a statistically significant difference was observed between the two groups in the rate of intraoperative diagnosis of HH (*p* = 0.0382) (Table 2). Conversion to open surgery, postoperative complications, or mortality was not observed in the two groups (Table 3). Mean hospital stay was 5.6 ± 1.3 days (range 3–8 days) and 5 ± 1.06 days (range 3–7 days) for groups A and B, respectively (*p* = 0.1511) (Table 3).

At mean follow-up of 33.2 ± 16.3 months (range 14–72 months) and 18.1 ± 11.3 months (range 1–38 months) (*p* = 0.0066) for groups A and B, respectively (Table 4), postoperative BMI was lower and %EBMIL was higher in group A as compared to group B, with a statistically significant difference (Table 4). Intergroup evaluation showed a highly statistically significant difference comparing the pre- and postoperative questionnaire scores in group B (Table 5). No statistically significant differences were observed comparing the pre- and postoperative GERD-HRQL mean scores between the two groups, although the difference (Δ) between the pre- and postoperative GERD-HRQL mean scores between the two groups was statistically significant in favor of patch placement (*p* = 0.0058) (Table 5).

Evaluating only the dysphagia by questionnaire items 7 and 8, mean preoperative scores were 1.58 ± 2.94 (range 0–8) and 2.53 ± 3.56 (range 0–12) for groups A and B, respectively (*p* = 0.4560). Mean postoperative scores were 0.92 ± 2.87 (range 0–10) and 0.88 ± 1.90 (range 0–7) for groups A and B, respectively (*p* = 0.9693). The Δ between pre- and postoperative mean dysphagia scores in the two groups was statistically significant in favor of patch placement (*p* = 0.0498).

Symptoms of de novo GERD were observed in one group A patient (GERD-HRQL score: preoperative 0, postoperative 13) and in one group B patient (GERD-HRQL score: preoperative 0, postoperative 12). HH recurrence was observed in two group A patients (16.6%) who underwent revisional laparoscopic gastric bypass between one and two years after LSG for severe GERD symptoms confirmed by pH manometry testing (postoperative GERD-HRQL scores 42 and 35, resp.). In one of these two patients, severe GERD symptoms significantly increased after pregnancy. HH recurrences were not observed in group B.

### 3.2. Discussion

The treatment of choice in obese patients with GERD symptoms is laparoscopic Roux-en-Y gastric bypass [7, 8]. However, patients' demand is more often in favor of laparoscopic sleeve gastrectomy (LSG), which in the patients' opinion is felt to be associated with lower morbidity, and it does not require long-term vitamins and elemental nutrient supplementation; the cost of which has to be covered by the patients themselves. However, LSG cannot be proposed to obese patients with severe GERD or to sweet eaters, and LRYGB has proven to be an excellent option in these cases [23]. The first LRYGB in the authors' unit was performed in March 2016, and since then, nine patients have undergone this procedure. Hiatoplasty with patch placement was performed in four out of these nine patients. The indication to perform LRYGB was in obese patients with GERD symptoms and/or sweet eating habits.

The present study is a retrospective analysis of two consecutive series of morbidly obese patients with HH who underwent LSG and one of the two types of hiatal closure, namely, simple posterior cruroplasty or posterior cruroplasty reinforced by a synthetic patch. Cruroplasty with mesh buttressing was associated with better results in terms of GERD symptoms control as compared to cruroplasty alone. The two groups were identical in terms of preoperative anthropometric characteristics and GERD symptoms, and patient allocation to the two arms of the study was based only on absorbable mesh availability in the hospital.

Reinforced cruroplasty during LSG in patients with large hiatal hernia has been proposed to prevent recurrence, with good results [16]. In the present series, the esophageal hiatus area measured ≤ 4 cm^2^. The authors hypothesized that a reinforced cruroplasty with absorbable patch during LSG might provide better results in terms of prevention of HH recurrence and GERD symptoms control, as compared to simple posterior cruroplasty, even in patients with smaller hiatal hernias. This hypothesis was based on the observation that in obese patients the diaphragmatic crura are substantially weakened, particularly the right bundle of the right crus, even if the esophageal hiatus area is not widely dilated. For the purpose of the present study, patients with an esophageal hiatus area measuring more than 4 cm^2^ were excluded from the analysis.

In the two series being consecutive, postoperative BMI was lower and %EBMIL was higher in the group with the longer duration of follow-up (group A), as expected. Quite surprising, instead, was the highly statistically significant difference that was observed comparing the pre- and postoperative questionnaire scores in group B that is the group with the higher BMI and the lower %EBMIL (Table 5). The fact that this effect was observed in patients with as yet less than optimal average weight control lends credit to the hypothesis that buttressing the crura might indeed be beneficial for GERD control in these patients, since this was the only differing variable. This result seems to contradict the hypothesis that the improvement of GERD symptoms after LSG alone might be related to the consequences of weight loss, such as decreased intra-abdominal pressure, or other factors including accelerated gastric emptying and reduced gastric acid secretion [24, 25]. Based on the present study, in fact, it seems that a factor contributing to the postoperative improvement of GERD symptoms after LSG might be the technique of hiatal closure.

In the authors' experience, the increase in HH repair during LSG was related to the recommendation reported in the literature suggesting a more aggressive approach towards the identification of any HH, and its repair when this is present [15].

The results of simple cruroplasty during LSG are still controversial [1, 26, 27]. Soricelli et al. reported a study in which 97 patients treated by LSG and posterior cruroplasty were compared to 281 patients who underwent LSG alone [1]. At a mean follow-up of 18 months, evaluating remission, improvement, persistence, and de novo GERD symptoms, better results were observed in patients undergoing HH repair [1]. An improvement of GERD symptoms at 6 and 12 months of follow-up was confirmed also by Daes et al. in a cohort of 134 patients [26]. Instead, Santonicola et al. reported a series of 78 patients who underwent LSG with concomitant HH repair by posterior cruroplasty and no significant decrease in GERD symptoms was observed at a mean follow-up of 14.6 months [27].

In the literature, few studies on HH repair with mesh reinforcement are reported [16, 18]. Most papers report the results of simple anterior or posterior cruroplasty [1, 26, 27]. El Chaar et al. reported a study in which mesh was placed at surgeons' discretion after posterior cruroplasty in case of HH measuring > 3 cm^2^, showing an improvement of postoperative GERD symptoms with or without mesh placement [18]. Ruscio et al. compared simple versus reinforced hiatoplasty, in patients with a hiatus area measuring ≤ 4 cm^2^ versus 4–8 cm^2^, respectively [16]. Notwithstanding the different hiatus sizes in the two groups, the patients who underwent LSG and reinforced hiatoplasty had better results in terms of GERD symptom control at 19 months after surgery, as compared to patients who underwent simple cruroplasty [16]. In this study, recurrent GERD was nil [16].

Another important aspect is that complications related to mesh placement were not observed in the present series nor in any other published one regarding hiatal closure during bariatric procedures [16, 18, 28]. The mesh and tacks employed in this series are absorbable, and no residual material in the hiatus area is observed at one year after surgery [16]. The mesh is absorbed by the body and replaced 1 : 1 with the patient's own type I collagen. In the literature, the use of absorbable mesh is associated with the absence of mesh-related complications [17, 29–31] as compared to nonabsorbable mesh placement for which mesh-related complications range between 1.3% and 20% [32–34].

The weaknesses of the present study are its retrospective nature, the small sample size of both groups, the lack of a standardized time of questionnaire administration, and the lack of preoperative pH-manometry data before LSG for more objective assessment of GERD. Moreover, the follow-up duration of group B is not long enough to draw definitive conclusions regarding long-term GERD outcomes. Finally, the sensitivity and specificity of questionnaires to detect GERD are reported to range between 65% and 75% [35, 36].

## 4. Conclusions

In conclusion, in patients with HH measuring up to 4 cm^2^, hiatal closure with or without mesh placement to reinforce the esophageal hiatus during LSG is both feasible and safe. Postoperative GERD symptoms improve with both techniques, but the improvement is highly statistically significant only with the use of mesh (*p* = 0.0002). Moreover, the difference (Δ) between pre- and postoperative GERD-HRQL mean scores in the two groups was statistically significant (*p* = 0.0058) and in favor of patch placement. In any case, a longer follow-up duration in group B is needed to confirm these data.

According to the available evidence, LSG with simple cruroplasty in patient with symptomatic severe GERD is not indicated, and LRYGB is the treatment of choice in these patients. In patients with mild/moderate reflux symptoms, however, LSG with cruroplasty and patch placement showed interesting postoperative GERD outcomes in the present series, taking into account also the frequent patients' demand to receive a LSG rather than a gastric bypass. Further studies including pH manometry evaluation, larger patients' series with longer follow-up duration, and a randomized study design are required to better evaluate these results.

## Figures and Tables

**Table 1:** Patients' characteristics.

	Group A *n* = 12	Group B *n* = 17	*p* value
Sex ratio (F : M)	10 : 2	13 : 4	1.0000
Mean age ± SD, years (range)	46.4 ± 11 (32–65)	48.4 ± 9.2 (31–63)	0.5918
Preoperative BMI ± SD, kg/m^2^ (range)	42.1 ± 8.3 (35–61)	43.5 ± 4.7 (35–51)	0.5757
T2DM, *n* (%)	6 (50)	11 (64.7)	0.4713
Hypertension, *n* (%)	7 (58.3)	8 (47.05)	0.7104
Sleep apnea syndrome, *n* (%)	9 (75)	11 (64.7)	0.6942
Mild–moderate, *n* (%)	6 (50)	7 (63.6)	0.7163
Severe needing CPAP^, *n* (%)	3 (25)	4 (23.5)	1.0000
Previous bariatric surgery, *n* (%)	0	0	1.0000

SD: standard deviation; BMI: body mass index; T2DM: type 2 diabetes mellitus. ^: continuous positive airway pressure.

**Table 2:** Symptoms, endoscopy, and biopsy findings.

	Group A *n* = 12	Group B *n* = 17	*p* value
GERD symptomatic patients, *n* (%)	8 (66.6)	9 (52.9)	0.7032
Preoperative diagnosis of HH, *n* (%)	6 (50)	15 (88.2)	**0.0382** ^∗^
Intraoperative diagnosis of HH, *n* (%)	6 (50)	2 (11.7)	**0.0382** ^∗^
Preoperative grade A esophagitis, *n* (%)	4 (33.3)	4 (23.5)	0.6828
Eradicated *Helicobacter pylori*, *n* (%)	3 (25)	8 (47.05)	0.2732

GERD: gastroesophageal reflux disease; HH: hiatal hernia. ^∗^Statistically significant differences in bold.

**Table 3:** Postoperative results.

	Group A *n* = 12	Group B *n* = 17	*p* value
Mean operative time ± SD, min (range)	195.4 ± 51.9 (130–300)	184.3 ± 39.09 (130–300)	0.5326
Conversion, *n* (%)	0	0	1.0000
Complications, *n* (%)	0	0	1.0000
Leaks, *n* (%)	0	0	1.0000
Associated procedures, *n* (%)	5 (41.6)	3 (17.6)	0.2180
Cholecystectomy	4 (33.3)	3 (17.6)	0.4029
Intraoperative ERCP/ES^	1 (8.3)	0	0.4138
Mean hospital stay ± SD, days (range)	5.6 ± 1.3 (3–8)	5 ± 1.06 (3–7)	0.1511
Mortality, *n* (%)	0	0	1.0000

SD: standard deviation. ^: endoscopic retrograde cholangiopancreatography/endoscopic sphincterotomy.

**Table 4:** Mean follow-up, pre- and postoperative BMI, and %EBMIL for each group.

	Group A *n* = 12	Group B *n* = 17	*p* value
Mean follow-up ± SD, months (range)	33.2 ± 16.3 (14–72)	18.1 ± 11.3 (1–38)	**0.0066** ^∗^
Postoperative BMI ± SD, kg/m^2^ (range)	29.7 ± 4.1 (22–36)	32.8 ± 3.2 (26–40)	**0.0317** ^∗^
%EBMIL ± SD (range)	74.1 ± 23.1 (39–118)	57.8 ± 15.7 (31–88)	**0.0315** ^∗^

SD: standard deviation; BMI: body mass index; EBMIL: excess body mass index loss. ^∗^Statistically significant differences in bold.

**Table 5:** GERD-HRQL questionnaire score.

	Group A *n* = 12	Group B *n* = 17	*p* value
Mean preoperative GERD-HRQL score ± SD (range)	16.5 ± 16.6 (0–45)	17.7 ± 14.1 (0–45)	0.8379
Mean postoperative GERD-HRQL score ± SD (range)	9.5 ± 14.6 (0–42)	2.4 ± 4.7 (0–15)	0.0712
*p* value	0.2793	**0.0002** ^∗^	
Δ mean GERD-HRQL score ± SD	−7 ± 2	−15.3 ± 9.4	**0.0058** ^∗^

SD: standard deviation. ^∗^Statistically significant differences in bold. Δ: difference.
